# Comparison of imputation methods for handling missing covariate data when fitting a Cox proportional hazards model: a resampling study

**DOI:** 10.1186/1471-2288-10-112

**Published:** 2010-12-31

**Authors:** Andrea Marshall, Douglas G Altman, Roger L Holder

**Affiliations:** 1 Centre for Statistics in Medicine, University of Oxford, Oxford, UK; 2 Warwick Clinical Trials Unit, University of Warwick, Coventry, UK; 3 Department of Primary Care Clinical Sciences, University of Birmingham, Birmingham, UK

## Abstract

**Background:**

The appropriate handling of missing covariate data in prognostic modelling studies is yet to be conclusively determined. A resampling study was performed to investigate the effects of different missing data methods on the performance of a prognostic model.

**Methods:**

Observed data for 1000 cases were sampled with replacement from a large complete dataset of 7507 patients to obtain 500 replications. Five levels of missingness (ranging from 5% to 75%) were imposed on three covariates using a missing at random (MAR) mechanism. Five missing data methods were applied; a) complete case analysis (CC) b) single imputation using regression switching with predictive mean matching (SI), c) multiple imputation using regression switching imputation, d) multiple imputation using regression switching with predictive mean matching (MICE-PMM) and e) multiple imputation using flexible additive imputation models. A Cox proportional hazards model was fitted to each dataset and estimates for the regression coefficients and model performance measures obtained.

**Results:**

CC produced biased regression coefficient estimates and inflated standard errors (SEs) with 25% or more missingness. The underestimated SE after SI resulted in poor coverage with 25% or more missingness. Of the MI approaches investigated, MI using MICE-PMM produced the least biased estimates and better model performance measures. However, this MI approach still produced biased regression coefficient estimates with 75% missingness.

**Conclusions:**

Very few differences were seen between the results from all missing data approaches with 5% missingness. However, performing MI using MICE-PMM may be the preferred missing data approach for handling between 10% and 50% MAR missingness.

## Background

Arbitrary missingness in covariates is common in prognostic modelling studies [1]. Many approaches for handling missing covariates when fitting a Cox proportional hazards model have been proposed such as likelihood based techniques (e.g. [2]) and imputation approaches (e.g. [3-5]). Likelihood based approaches generally require problem-specific programmes and therefore are not generally readily available. The best imputation approach remains unclear. A simulation study [6] comparing imputation procedures suggested that performing multiple imputation (MI) with regression switching (MICE) and using predictive mean matching (PMM) [5] may be preferred over other MI approaches or single imputation (SI) with highly skewed incomplete continuous covariates. In addition, MICE was found to produce similar results to MI using data augmentation and assuming a joint multivariate normal model or a general location model [5]. It is not clear whether MICE with PMM would remain beneficial in other populations, where the data may be closer to the underlying assumptions of the imputation methods.

Simulation studies based on fully generated data may be criticised for being too simplistic as they often use models based on limited perceived structures of the population to generate the datasets thus not always fully reflecting a realistic population even if based on attributes from real datasets. A resampling study, however, samples data from a large empirical complete dataset. The data in the smaller samples are observations from real patients [7] and thus reflect the appropriate level of diversity and variability found in realistic populations [8]. The initial dataset needs to be sufficiently large to permit numerous samples of reasonable size to be selected without seriously endangering any future assumption of independence; it can be from one large study (e.g. [9]) or the combination of several similar studies (e.g. [10]). In addition, for prognostic modelling studies, an adequate number of events, is considered necessary to provide stable conclusions within the smaller samples, with a general rule of thumb of at least ten events per covariate studied [11]. Sampling with replacement, as in bootstrapping [12], replaces selected cases back into the potential selection pool after each draw [13]. The variability between samples is similar to what would be experienced among many samples from an infinite population [14]. Alternatively, sampling without replacement allows each case to be selected only once for a particular sample [13]. Sampling without replacement is only suitable when the available population can be considered infinitely large, and thus representative of the true population, or when the maximum sample size required is less than 10% of the total population [9].

This paper presents the results of a resampling study to investigate the effects of different methods used to handle multivariate missing covariate data when fitting a Cox proportional hazards model to the full set of covariates.

## Methods

### Resampling Population

Baseline data from a large randomised colorectal cancer trial [15,16] formed the empirical population for this resampling study. Approval from the Chief Investigators of this trial was granted for use of their data in this resampling study. Data were available on a total of 7507 patients randomised between May 1994 and September 2003 to assess benefit of adjuvant chemotherapy (CT) in terms of overall survival. The collection of eight patient, tumour and planned treatment characteristics was mandatory at randomisation and hence all were complete (Table 1). The randomised treatment for each patient was unavailable for this research. The exclusion of treatment is not detrimental to this resampling study as its purpose was to assess the impact of enforcing missing data on an obtained prognostic model for all patients irrespective of the randomised treatment.

**Table 1:** Summary of the data and characteristics of the colorectal cancer trial patients

Characteristic	Label	Level	N(%)
Age (years)	Age	Median (IQR)	62 (55-68)
		Mean(SD)	61 (9.95)

Sex	Sex	1 = Female	3013(40%)
		2 = Male	4494(60%)

Site of Cancer	Site	0 = Colon only	5197(69%)
		1 = Rectum/both	2310(31%)

Stage	Stage	0 = Dukes' A/B	3775(50%)
		1 = Dukes' C	3732(50%)

Pre-operative	PRE-RT	1 = No	7147(95%)
RT		2 = Yes	360(5%)

Post-operative RT	POST-RT	1 = No	6511(87%)
planned		2 = Yes	996(13%)

Indication for	CT-INDIC	1 = Clear	4320(58%)
CT		2 = Uncertain	3187(42%)

CT Schedule	CT-SCH	1 = Every week	3757(50%)
		2 = Every 4 weeks	3750(50%)

**Key**: IQR = Inter-quartile range, SD = standard deviation, RT = radiotherapy, CT = Chemotherapy

The distribution of age was unimodal but modestly skewed towards a more elderly population (skewness = -0.67). Most covariates were weakly associated with each other, but stage of disease and indication for CT were highly correlated (phi correlation coefficient (r) = -0.72), whilst site of cancer was moderately correlated with pre-operative radiotherapy (RT) (r = 0.32) and planned post-operative RT (r = 0.42).

Follow-up information was available until October 2003, by which time there had been 2652 (35%) events among the 7507 patients. For the 4855 (65%) patients with censored observations, the median length of follow-up was 6.5 years with a maximum of 9 years. The Kaplan-Meier estimated survival probability at five years was 64%.

### Samples

Each dataset in the resampling study consisted of 1000 cases, which represented the average sample size from a review of published prognostic studies [1], and was sampled with replacement from the full colorectal dataset. The observed covariate data, survival time and event status from these sampled cases were utilised. Using simple random sampling allowed some variability in the covariate structure and the proportion of events whilst retaining, on average, the 65% censoring present in the whole of the colorectal dataset.

### Replications

A total of 500 replications were performed. With this number of replications, regression coefficients for six of the eight prognostic covariates could be estimated with at least 5% accuracy [17], given the coefficient values and associated standard errors (SEs) from fitting a Cox proportional hazards model including all eight covariates to the whole colorectal dataset. The regression coefficients for the CT schedule and site of cancer, which were non-significant in the model using the whole colorectal dataset, could be estimated with 13% and 37% accuracy respectively with 500 replications. An unworkable number of replications of approximately 3500 and 27000 replications respectively would be required to achieve 5% accuracy, as the regression coefficients were close to zero.

### Imposing missingness on multivariate data

Missingness was imposed on stage, post-operative RT and age according to seven missing data patterns (*R*_*i*_,*i *= 1,...,7) chosen to match those observed in an ovarian cancer study [18] (Table 2). In practice, age is unlikely to be missing, but was used for illustrative purposes to enable the effects of a continuous covariate with missing values to be investigated. Five overall rates of missingness, denoted *p*_0_, of 5%, 10%, 25%, 50% and 75% per case were considered to cover the range of missing data that may be seen in practice, such that *p*_0 _cases had at least one covariate with missing values.

**Table 2:** Details of the patterns of missingness in the ovarian cancer study 18

**Pattern (*R***_***i***_**)**	Stage	POST-RT	Age	**Frequency Probability (*p***_***i***_**)**	Cumulative probability
**0**	**1**	**1**	**1**		
**1**	**1**	**1**	0	0.08	0.08
**2**	**1**	0	**1**	0.17	0.25
**3**	**1**	0	0	0.04	0.29
**4**	0	**1**	**1**	0.25	0.54
**5**	0	**1**	0	0.04	0.58
**6**	0	0	**1**	0.34	0.92
7	0	0	0	0.08	1.00

% missing out of total incomplete cases	71	64	22		

**Key**: **1 **= observed and 0 = missing

Missingness was imposed using a missing at random (MAR) mechanism [19], where the missingness was associated with shorter survival times, having cancer of the rectum or both rectum and colon, having a clear indication for CT and the observed values of stage, post-operative RT and age. This MAR mechanism resulted in a higher proportion of missing observations among older cases, those with Dukes' C stage or those planning on having post-operative RT. The missing data patterns (Table 2) were generated using the procedures proposed by van Buuren et al [20], summarised in additional file 1, to give a total of *p*_0 _cases with at least one covariate with missing data.

### Missing data methods and imputation model

We investigated five missing data methods, for which code is freely available within the R statistical software (Table 3). These were complete case analysis (CC), single imputation (SI) using predictive mean matching [5], MI fitting separate flexible additive imputation models to each incomplete covariate with predictive mean matching [21] (MI-aregImpute), MI using regression switching (MI-MICE) and the addition of predictive mean matching (MI-MICE-PMM) [5]. Predictive mean matching incorporates a non-parametric element and therefore relies less on the parametric assumptions of the imputation models. The imputation models included all available covariates, the event status and the survival times after a logarithmic transformation. Ten imputations were performed for each of the MI approaches, which still gave a minimum relative efficiency compared to using an infinite number of imputations [22] of approximately 95% when 75% overall missingness was imposed. Each missing data method was applied to the same 500 independent samples generated.

**Table 3:** Details of the five missing data methods investigated

Label	Missing data method
CC	Complete case analysis

SI	Single imputation using regression switching imputation with predictive mean matching with only one imputation fitted using the 'pmm' function within the mice library [40]

MI-aregImpute	MI fitting flexible additive imputation models using the 'aregImpute' function in the Hmisc library [21]

MI-MICE	MI using regression switching imputation with linear or logistic regression models as appropriate for each incomplete covariate fitted using the mice library [40]

MI-MICE-PMM	MI using regression switching imputation with predictive mean matching fitted using the 'pmm' function within the mice library [40]

**Key**: PMM = predictive mean matching; MI = multiple imputation

### Analysis and outcomes of interest

The applicability of the linearity assumption for age was investigated using fractional polynomials [23], fitted using the 'fp' function within the 'mfp' library in the R statistical software [24]. The appropriate functional form for fitting the continuous covariate, age, in the regression model was assessed using fractional polynomials based on 500 full datasets, prior to missing data being imposed. The most commonly chosen functional form for age was linear; selected in 90% (n = 452) of samples. Therefore, age was fitted assuming a linear relationship throughout this resampling study.

A Cox proportional hazards model including all eight covariates was fitted to each dataset. The outcomes of interest were the regression coefficients, their associated SEs and the significance of each covariate in the regression model. The performance of the prognostic model in each dataset was assessed in terms of the Nagelkerke's R^2 ^statistic [25], the prognostic separation D statistic [26] and the 2 and 5 year predicted survival probabilities.

The regression coefficient estimates were compared against the "true" values in terms of their bias, coverage and efficiency [27]. The average regression coefficient estimates and associated empirical SE obtained from performing 20000 replications of 1000 cases with complete data were considered as the "true" values. This analysis produced SEs that were more representative of the resampling study to be performed than would have been obtained from fitting a Cox model to the available population of 7507 patients.

To incorporate the appropriate uncertainty from imputation, the results from each multiply imputed dataset were combined using Rubin's rules [22] after suitable transformations to approximate normality, as previously recommended [28]. The median and inter-quartile ranges of the Nagelkerke's R^2 ^statistics were determined for each of the 500 replicated datasets [29]. Any deficiencies in these combining approaches should be similar across all MI methods, thus still allowing a valuable comparison. The outcomes of interest from the 500 replicated datasets were summarised using the average or median value where appropriate.

## Results

The average percentage of available covariate data items for the 1000 cases in each dataset remained relatively high for all amounts of missingness imposed; ranging from 99% with 5% missingness to 86% when 75% of cases had one or more missing data items.

### Regression coefficient estimates from a Cox proportional hazards model

**Figure 1:**
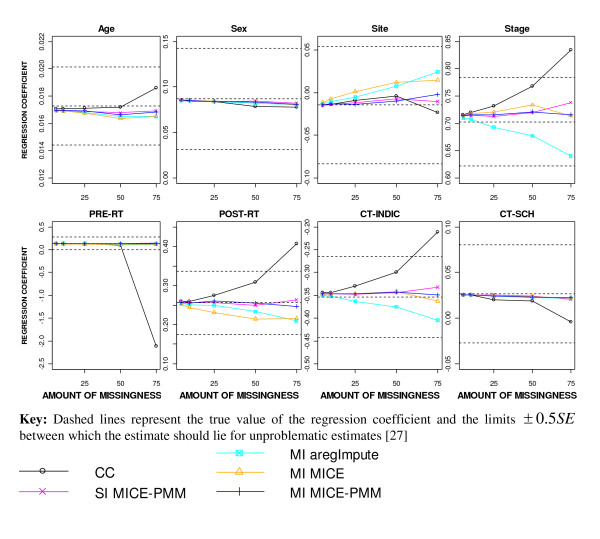
**Regression coefficient estimates after applying different missing data methods to increasing percentages of MAR missingness**.

**Figure 2:**
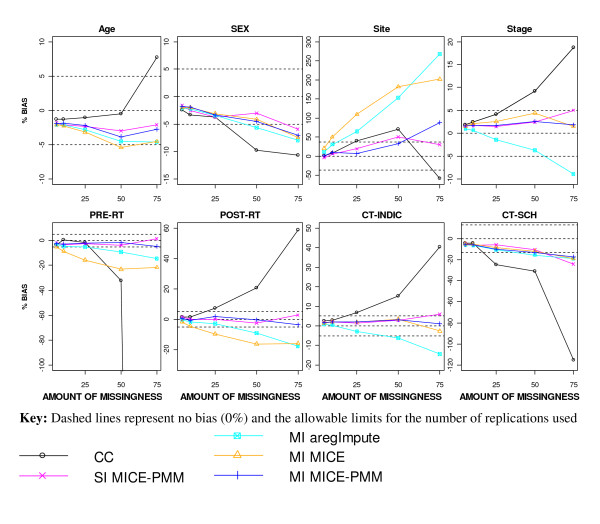
**Percentage bias in the regression coefficient estimates after applying different missing data methods to increasing percentages of MAR missingness**.

Using a complete case (CC) analysis produced very unstable regression coefficient estimates when there were large amounts of missingness, especially for the binary pre-operative RT covariate, which had a 95:5 split in the data. All estimates remained within the limits for unproblematic estimates [27] of ± 0.5*SE *from the true value with up to 50% missingness. Only the regression coefficient estimates for stage, pre-operative RT, post-operative RT and indication for CT (Figure 1) could be deemed problematic [27] with 75% missingness. However, the percentage biases were more extreme than the specified accuracy given the number of replications performed for the majority of covariates with 25% or more missingness (Figure 2). The exceptions were for stage, sex and age, where the bias remained within the specified 5% accuracy until at least 50% missingness.

After imputation, all regression coefficient estimates remained within ± 0.5*SE *of the true value for all levels of missingness (Figure 1). Using SI or MI-MICE-PMM produced the least biased estimates for all covariates (Figure 2). Greater percentage bias was seen for site, pre-operative RT and post-operative RT when using MI-MICE than with the other imputation approaches, producing biases greater than 5% with as little as 5% missingness. The estimates for stage and indication for CT were slightly more underestimated after MI using the "aregImpute" function (MI-aregImpute).

### SE of regression coefficient estimates

**Figure 3:**
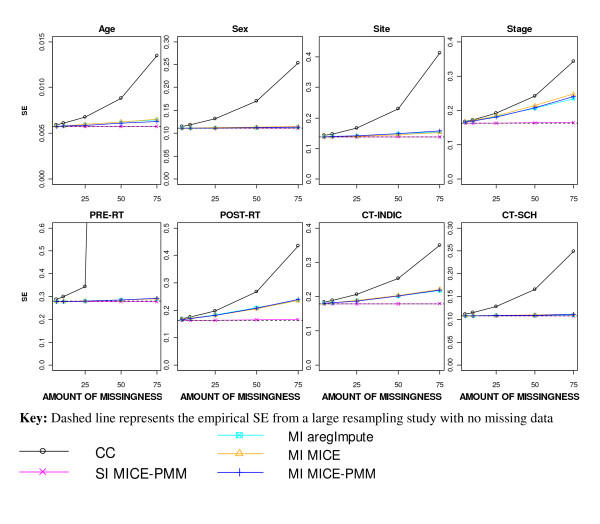
**SE estimates after applying different missing data methods to increasing percentages of MAR missingness**.

The average SE estimates from the different MI approaches were similar and, as expected, fell below the inflated SE estimates after a CC analysis and in general above the underestimated SE after SI (Figure 3). The SEs after a CC analysis were extremely unreliable for pre-operative RT. With increasing levels of missingness, the SE after MI increased more for the incomplete binary covariates than for the continuous covariate; age.

### Coverage

**Figure 4:**
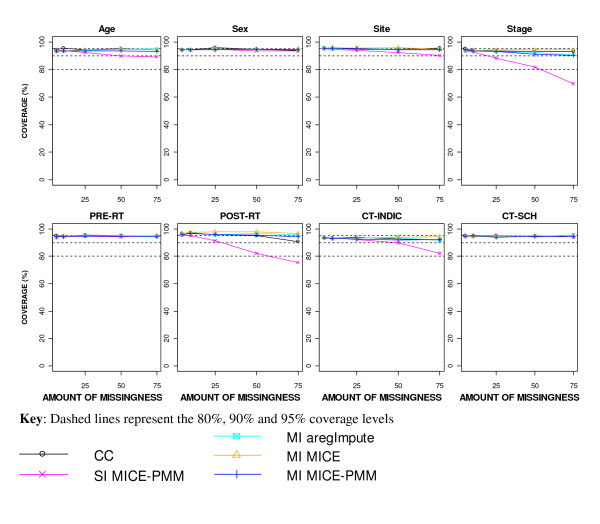
**Coverage after applying different missing data methods to increasing percentages of MAR missingness**.

Coverage was most affected using SI (Figure 4). The coverage after SI for stage and post-operative RT fell to around 90% when 25% of the cases were incomplete and below 80% with 75% missingness. The coverage of indication for CT fell to around 80% using SI with 75% missingness. The coverage for the remaining five covariates for SI and all covariates using a CC analysis or applying MI remained around 90% for all levels of missingness.

### Significance of covariates in the prognostic model

**Figure 5:**
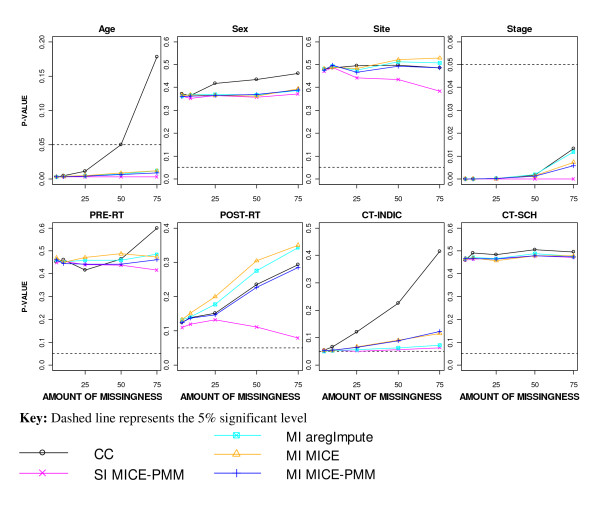
**Significance of the covariates in the prognostic model after applying different missing data methods to increasing percentages of MAR missingness**.

The two highly prognostic covariates of age and stage remained significant in the model even with 75% missingness using any missing data method, except when performing a CC analysis where age became non-significant at the 5% level with 50% or more missingness (Figure 5). The indication for CT was of borderline prognostic ability in the resampling study, but became non-significant after a CC analysis with 10% or more missingness and after imputation with 25% or more missingness. After SI, the non-prognostic covariates of site and post-operative RT became more significant in the model with higher levels of missingness, although always remaining non-significant. In contrast, post-operative RT became less significant in the model with increasing levels of missingness for all the MI approaches.

### Model performance measures

**Figure 6:**
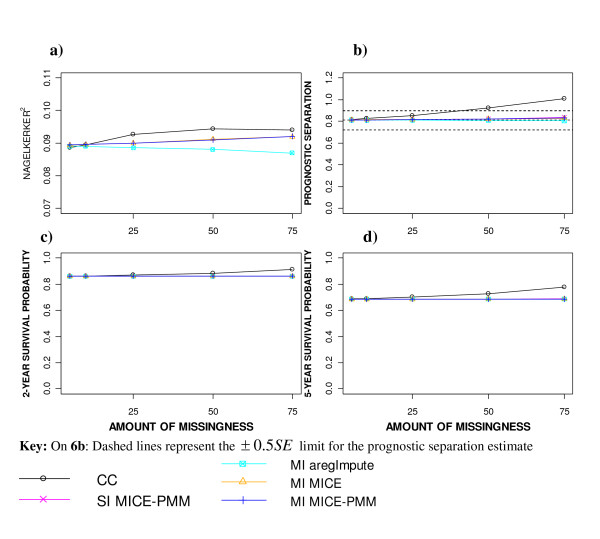
**Model performance measures after applying different missing data methods to increasing percentages of MAR missingness a) Nagelkerke R^2 ^statistic, b) Prognostic separation D statistic and c d) Predicted 2 and 5 year survival from Cox model, respectively**.

The Nagelkerke's R^2 ^statistic increased slightly with higher levels of missingness after performing a CC analysis, suggesting that the model had better predictive ability when fewer cases were analysed (Figure 6a). In contrast, applying MI-aregImpute produced slightly lower predictive ability with increasing levels of missingness.

Similar prognostic separation values were produced after imputation for all percentages of missingness imposed (Figure 6b). However when a CC analysis was performed, the prognostic separation statistic estimates were more than ± 0.5*SE *from the true value when the missingness exceeded 25% missingness.

The predicted survival probabilities were unaffected by the amount of missingness or the imputation approach applied (Figure 6c and 6d). However, the predicted survival probability estimates after performing a CC analysis were consistently higher than those obtained after imputation and diverged further away as the level of missingness increased, reflecting that the incomplete cases were associated with survival.

## Discussion

This resampling study used a large complete empirical dataset as the population from which samples were drawn. Hence, the distributions for the survival times and the covariates reflected those seen in a real situation. Empirical evidence from an ovarian cancer study [18] provided realistic patterns of missingness and the relative proportions of missing values for each incomplete covariate.

This resampling study identified that, with up to 10% multivariate MAR missingness, a CC analysis provided reasonable estimates of the regression coefficients, associated SEs, significance of the covariates in the model and model performance measures. However, these measures were all adversely affected when there were 25% or more incomplete cases. These findings corroborate the results seen by others with univariate missingness ([30]; [31]) and with multivariate missingness [6], although they obtained unbiased regression coefficients estimates with a MAR mechanism, as the mechanism that depended on outcome was imposed on the covariate with the least amount of missingness only. These results suggest that a CC analysis with 10% or less missingness is useful provided that the missing data mechanism is not highly dependent on outcome, especially at shorter survival times ([32]; [33]), the sample size is reasonably large [31] and the hazard ratios for survival are not large [33]. In practice, the missing data mechanism is rarely fully known and so the dependence on survival will be unclear and therefore in general CC analysis should be avoided. Caution is needed when covariates have very uneven splits with only a small proportion of cases in one group, e.g. pre-operative RT, as this can lead to very unstable regression estimates and SEs when performing a CC analysis.

Using SI with PMM produced reasonable regression coefficient estimates that were within 20% of the true value for all covariates except the non-prognostic covariate site of cancer, where the bias reached 50% with 75% missingness. The underestimation of the variability and hence narrower confidence intervals after SI, however, resulted in poor coverage with 25% or more missingness, especially for the incomplete covariates. Therefore SI is not recommended for use with more than 10% MAR multivariate missingness, as previously found (e.g. [6]).

This resampling study identified that standard MI methods for handling missing covariate data can be adequately used in prognostic modelling studies where the outcome is survival time with up to 50% MAR missingness within binary or continuous covariates with moderate skewness. The distribution of the incomplete covariates can affect the performance of the MI approaches, as poorer results were seen in another simulation study with highly skewed covariate data [6]. With more than 50% MAR missingness, MI may produce biased and misleading results and therefore its use with this high level of missingness should be with caution and considered only as part of a sensitivity analysis.

MICE-PMM outperformed all other MI approaches considered in this resampling study with one moderately skewed covariate. This corroborated the findings from previous research, where MICE-PMM was also the preferred approach with highly skewed covariates ([6]; [34]; [35]; [36]). MICE-PMM proved empirically to be more useful than those with stronger distributional assumptions, despite its lack of formal theoretical justifications [37].

The performance of the imputation approaches depends on the consistency between the imputation and analysis models [38], the more compatible these models are the better the imputation methods will perform. MICE-PMM imputes data from observed cases with similar predictive values and therefore relies less on any distributional assumptions of the covariates and outcome and on the consistency of the imputation and analysis models compared to other MI approaches. Any biases that may occur after including log transformed survival time and event status in the imputation model and then using a Cox proportional hazards model to analyse the imputed datasets are generally smaller when MICE-PMM is used, This has resulted in an improved performance of MICE-PMM with a censored survival outcome and highly skewed covariates [6] but also in this resampling study with less skewed data.

However, MICE-PMM may not remain the better approach with more normally distributed incomplete covariates or with a fully observed normally distributed outcome, where the imputation and analysis models are more compatible. In addition, care must be taken when using MICE-PMM with small samples and when covariates have rare events, as there may not be many available cases to be used as imputed values. A better approach for including survival data in an imputation model may be using the Nelson-Aalen estimate of the cumulative hazard for survival [39].

These results broadly confirm previous findings, but they are only based on one realistic population and one multivariate MAR missing data mechanism. Therefore the results may not be fully generalisable to alternative populations, with differing distributions, correlations and missing data mechanisms.

## Conclusion

With 5% missingness, very few differences were seen between the results from performing a CC analysis, SI or MI using MICE-PMM. However, applying MI using MICE-PMM was found in this resampling study to be the most useful missing data approach for handling between 10% and 50% MAR missingness.

## Competing interests

The authors declare that they have no competing interests.

## Authors' contributions

AM participated in the design, coordination and analysis of this study and drafted the manuscript. DGA participated in the design of the study, the interpretation of the data and helped in the writing of the manuscript. RH advised on the design and interpretation of the study, and participated in the revision of the manuscript. All authors have read and approved the final manuscript.

## Pre-publication history

The pre-publication history for this paper can be accessed here:

http://www.biomedcentral.com/1471-2288/10/112/prepub

## Supplementary Material

Additional file 1 **Appendix**. Procedures for generating a multivariate missing at random mechanism Click here for file
