# Supplementary material for: Comparison of imputation methods for handling missing covariate data when fitting a Cox proportional hazards model: a resampling study
Source: BMC Med Res Methodol. 2010 Dec 31;10:112. doi: 10.1186/1471-2288-10-112 (PMC3019210; doi:10.1186/1471-2288-10-112)
Supplement: Additional file 1 — Appendix. Procedures for generating a multivariate missing at random mechanism [file 1471-2288-10-112-S1.PDF]

Appendix: Procedures for generating a multivariate missing at random mechanism

For each p_0 (p_0 = 5%, 10%, 25%, 50%, 75%), an arbitrary multivariate MAR missingness mechanism, according to the patterns ($R_i, i = 1,...,7$) given in Table 2, was imposed using the following procedures, proposed by van Buuren et al [17], to give a total of p_0 cases with at least one missing value.

1. Each case was randomly assigned to one of the seven possible missing data patterns ($R_i, i = 1,...,7$) given in Table 2 with specified probability (p_i, $i = 1,...,7$), by comparing an assigned random value from the Uniform distribution with the cumulative probability of being in each pattern.
2. Within the sample allocated to a particular pattern, R_i, $i = 1,...,7$
   a. A linear score was calculated for each case using the observed values for the variables related to the missingness and the associated regression weights for the required pattern (R_i). The regression weights for the linear score were based on the regression coefficients from fitting a linear or logistic regression model, as appropriate, to each of the incomplete covariates with all other variables associated with the missingness as covariates and using the whole colorectal dataset. For patterns involving more than one incomplete covariate, the sum of the regression coefficients was used.
   b. The cases were divided into three subgroups, $j = 1,2,3$, using the 33% and 66% percentile values of their linear scores as cutpoints, with a sample size of n_j cases within the jth each subgroup size.

c. The odds (O_j) of having R_i in each subgroup compared to the reference subgroup with the lowest linear scores were specified as linearly increasing, such that the second and third subgroups had double and treble the odds of the reference subgroup.

d. The probability of having R_i was calculated for each case as

$$\frac{1000 \times p_0 p_i O_j}{n_j \sum_{j=1}^{3} O_j},$$

e. Each case was then assigned another random value drawn from the Uniform distribution and if this value did not exceed their calculated probability then the data for that case were set to be missing according to the appropriate missing data pattern, R_i.
